# Multi-modality imaging-monitored creation of rat orthotopic pancreatic head cancer with obstructive jaundice

**DOI:** 10.18632/oncotarget.17347

**Published:** 2017-04-21

**Authors:** Zhibin Bai, Yaoping Shi, Jianfeng Wang, Longhua Qiu, Gaojun Teng, Feng Zhang, Xiaoming Yang

**Affiliations:** ^1^ Image-Guided Biomolecular Intervention Research, Section of Interventional Radiology, Department of Radiology, University of Washington School of Medicine, Seattle, WA, USA; ^2^ Department of Radiology, Zhongda Hospital, Southeastern University, Nanjing, China; ^3^ Department of Radiology, Sir Run Run Show Hospital, Zhejiang University School of Medicine, Hangzhou, China

**Keywords:** pancreatic cancer, multi-modality imaging, model, rat, orthotopic

## Abstract

**Purpose:**

To investigate the feasibility of using multi-modality imaging to monitor the creation of rat models with orthotopic pancreatic head cancer with obstructive jaundice.

**Results:**

27 of 52 rats (51.92%) developed pancreatic head cancer. The tumor formation rate was significantly higher in the animal group receiving bioluminescent tumor, compared to the group receiving non-bioluminescent donor tumors [78.1% (25/32 rats) vs 10.0% (2/20 rats), *P* = 0.0001]. Both ultrasound imaging and MRI clearly characterized the orthotopic tumors. Laboratory biochemistry test for those rats with obstructive jaundice showed elevated levels of bilirubin, aspartate transaminase (AST), alkaline phosphatase (ALT) and gamma-glutamyl transpeptidase (λ-GGT), compared with those rats without jaundice (*P* < 0.05). Correlative pathology confirmed that all tumors were ductal adenocarcinomas, and located in pancreatic head regions.

**Materials and Methods:**

Rat pancreatic adenocarcinoma cells (DSL-6A/C1) were first transfected with lentivirus/mCherry-luciferase genes, and then subcutaneously implanted into flanks of donor immunocompetent Lewis rats, to create pancreatic tumor tissues. The tumor tissues from donor rats with either bioluminescence signal or without the signal were then transplanted into the pancreatic heads of 52 recipient Lewis rats. Bioluminescence optical and ultrasound imaging, as well as magnetic resonance imaging (MRI), were performed to follow up the tumor formation and growth in these tumor-transplanted rats. Physical examination and biochemistry test were used to discern the rats with obstructive jaundice. The rats were euthanized for subsequent histologic correlation and confirmation.

**Conclusions:**

We successfully created a new rat model with orthotopic pancreatic head cancer, which can be accurately monitored and visualized by different imaging modalities.

## INTRODUCTION

Pancreatic cancer is one of the most lethal and aggressive human malignancies. It is the fourth most frequent cause of cancer-related deaths in the world, with an approximately 37,000 deaths in the United States in 2012 and 50,000 deaths per year in Western Europe [1, 2]. At its advanced stage, the disease is highly resistant to chemotherapeutics such as gemcitabine and 5-fluorouracil. Although surgical resection offers the best long-term survival, at the time of diagnosis radical surgery is possible in only 5–25% of patients, with a 5-year survival rate no more than 29% [3, 4].

A reproducible preclinical animal model with pancreatic cancer is the critical necessity in studying the underlying causes of tumor development, growth and dissemination of pancreatic cancer, as well as developing the effective and novel treatment for this deadly malignancy [5–8]. Currently, the most widely used animal model of pancreatic cancer was created by subcutaneously or orthotopically implanting xenografts of human tumor cells into nude mice [9]. The implanted human pancreatic cancer xenografts can grow to a substantial tumor mass in the immunocompromised or severe combined immunodeficiency (SCID) mice within a relatively short period, which presents some values in preclinical studies to explore the alternative diagnosis and treatment methods for pancreatic cancers. However, such models cannot reflect the interaction between the tumor and a fully competent immunologic system of the tumor host. Genetically engineered mouse models (GEMM) of pancreatic cancers are helpful in advancing our understanding of the genetic pathways responsible for initiating and driving progression of the disease. Genetically engineered mice are created by interbreeding mice with different phenotypes, such as LSL-Kras^G12D/+^, LSLTrp53R^172H/+^ and Pdx-1-cre (KPC), and can spontaneously develop pancreatic cancers. KPC-derived pancreatic tumors appear to more closely mimic the pathophysiologic characteristics of human pancreatic ductal adenocarcinoma (PDAC), recapitulating many of the clinical features, histopathology, therapeutic resistance, and invasive nature of the human PDAC that is not consistently observed in other xenograft models. However, these mouse models of pancreatic cancers are not suitable for the studies of developing the imaging-guided ablative therapies, such as interstitial radiofrequency ablation [1, 10, 11], microwave ablation [12, 13], laser ablation [14] and irreversible electroporation ablation [15], due to the tiny size of the pancreas of a mouse that does not fit in the requirement for placement of the clinically-used ablation electrodes.

In addition, obstructive jaundice is still recognized as the primary complication in patients with ductal adenocarcinoma of the pancreatic head, and occurs in approximately 75% of this population [16, 17]. Obstructive jaundice is associated with the significantly decreased survival of patients with pancreatic head cancer [16, 17]. Thus, it is essential to create an animal model of orthotopic pancreatic head cancer with obstructive jaundice, which can be used for developing new strategies of imaging diagnosis and imaging-guided interventional treatment for pancreatic cancer [6]. Molecular imaging allows the visualization of normal and abnormal pathophysiologic events in living subjects at the molecular or genomic level rather than at the anatomic level [18, 19]. Therefore, in this study, we attempted to fully apply the advantages of molecular imaging technology in assisting the creation of rat orthotopic pancreatic head cancer with obstructive jaundice.

## RESULTS

**Figure 1:**
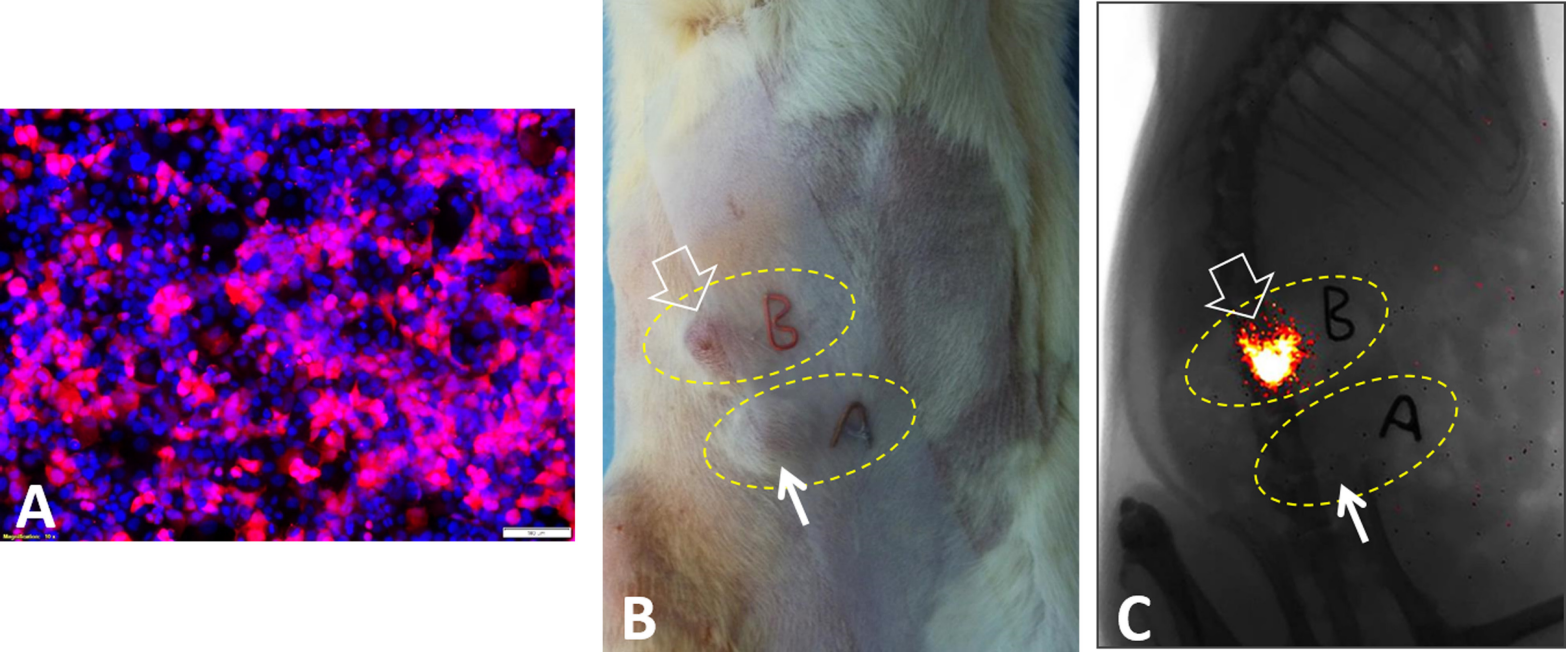
(**A**) Fluorescent microscopy shows successful labeling of pancreatic cancer cells by luciferase/m-cherry/lentivirus, which leads to pink-color light emission after administration of luciferin. (**B** and **C**) After subcutaneous implantation of the luciferase-labeled pancreatic cancer cells in donor animals, we perform optical imaging to screen bioactive or metabolic active pancreatic cancer tissues, i.e. “bright” tumor tissues that emit luciferase light (open arrows, C), compared to non-bioactive “dark” tumor tissues (short arrows, C). Thus, optical imaging enables us to “sort out” bioactive pancreatic cancer tissues for the next step, orthotopic transplantation in recipient animals.

Orthotopic implantation of five tumor fragments into the pancreatic head of each recipient rat was successfully performed in 52 Lewis rats, among which 20 rats received the donor tumor pieces without visible bioluminescence signals (no signals) and 32 rats received the tumor pieces with apparent bioluminescence signals (bright signals). The tumor formation rate in pancreatic head was significantly higher in the animal group receiving bioluminescent tumor tissues (i.e. bio-active or metabolic active tumor tissues), compared to the group receiving non-bioluminescent donor tumors [78.1% (25/32 rats) vs 10.0% (2/20 rats), *P* = 0.0001] (Figure 1).

**Figure 2:**
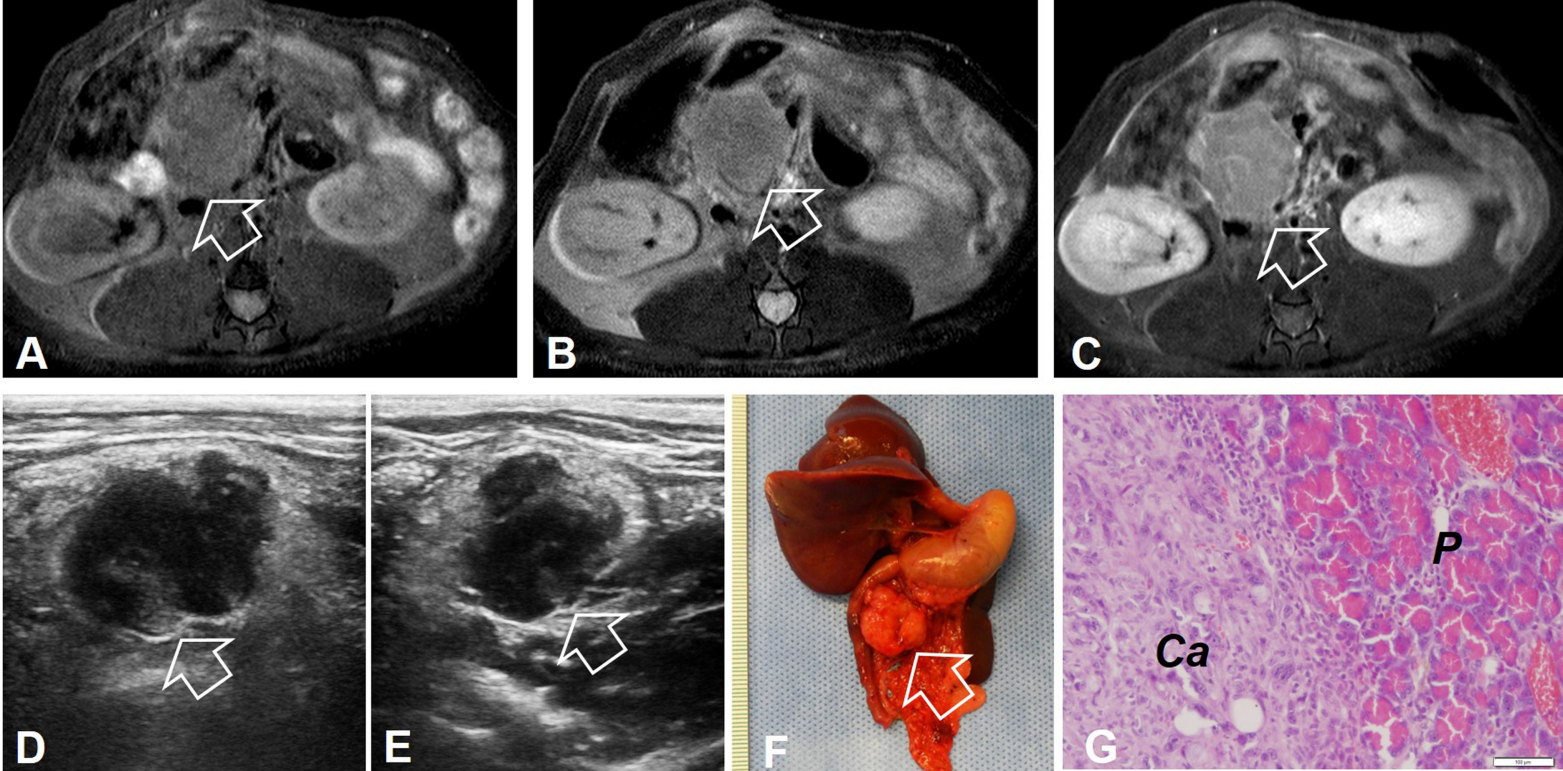
title (**A–C**) MR images of the orthotopic pancreatic head cancer of a recipient rat. The tumor appears as homogeneous hypointense signal on axial T1WI (arrow on A), homogeneous hyperintense signal on T2WI (arrow on B), and heterogeneous enhancement on contrast-enhanced T1WI (arrow on C). (**D** and **E**) Ultrasound imaging demonstrates the tumor mass as inhomogeneously hypoechoic intensity on the transvers and longitudinal images (arrows). (**F**) Photograph of the gross specimen shows a typical pancreatic head tumor (arrow). (**G**) Histology with H&E staining confirms the formation of the pancreatic ductal carcinoma (Ca) and the normal pancreas (P).

MR imaging showed irregular-shaped mass in the pancreatic head, demonstrated as homogeneous hypointensity on T1WI, hyperintensity on T2WI, and homogeneous enhancement 30 seconds after the intravenous administration of MR contrast agent, compared with the pre-enhancement T1WI (post- vs pre-enhancement signal intensity = 1261.9 ± 139.8 vs. 986.2 ± 114.9, *P* = 0.002) (Figure 2A–2C). For 6-week imaging follow-up of the animal group receiving bioluminescent active donor tumor tissues, the tumor grew from the average volume of 2.14 ± 0.21 cm^3^ to 4.13 ± 0.15 cm^3^, with an average growth rate of 0.49 ± 0.11 cm^3^ per week. On the ultrasound images, the tumors were shown as multilobulated inhomogeneous hypoechoic masses in the regions of pancreatic heads (Figure 2D and 2E). However, due to the deep-seated location of tumors in the abdominal cavity, which rendered the distance between the tumor and the skin beyond the penetration depth of bioluminescence, the majority of tumors were not visible in the bioluminescent optical images.

Pathologically, 7 (26%) of 27 animals with tumors developed disseminated metastatic foci in the retroperitoneum, greater omentum, mesentery, and intestines at the end of the 6-week follow up post-tumor implantation. Three (15%) of 27 rats developed bloody and malignant ascites with obvious sign of cachexia. Microscopically, the tumors displayed as a moderately to well differentiated ductal pancreatic carcinomas (Figure 2F and 2G).

**Figure 3:**
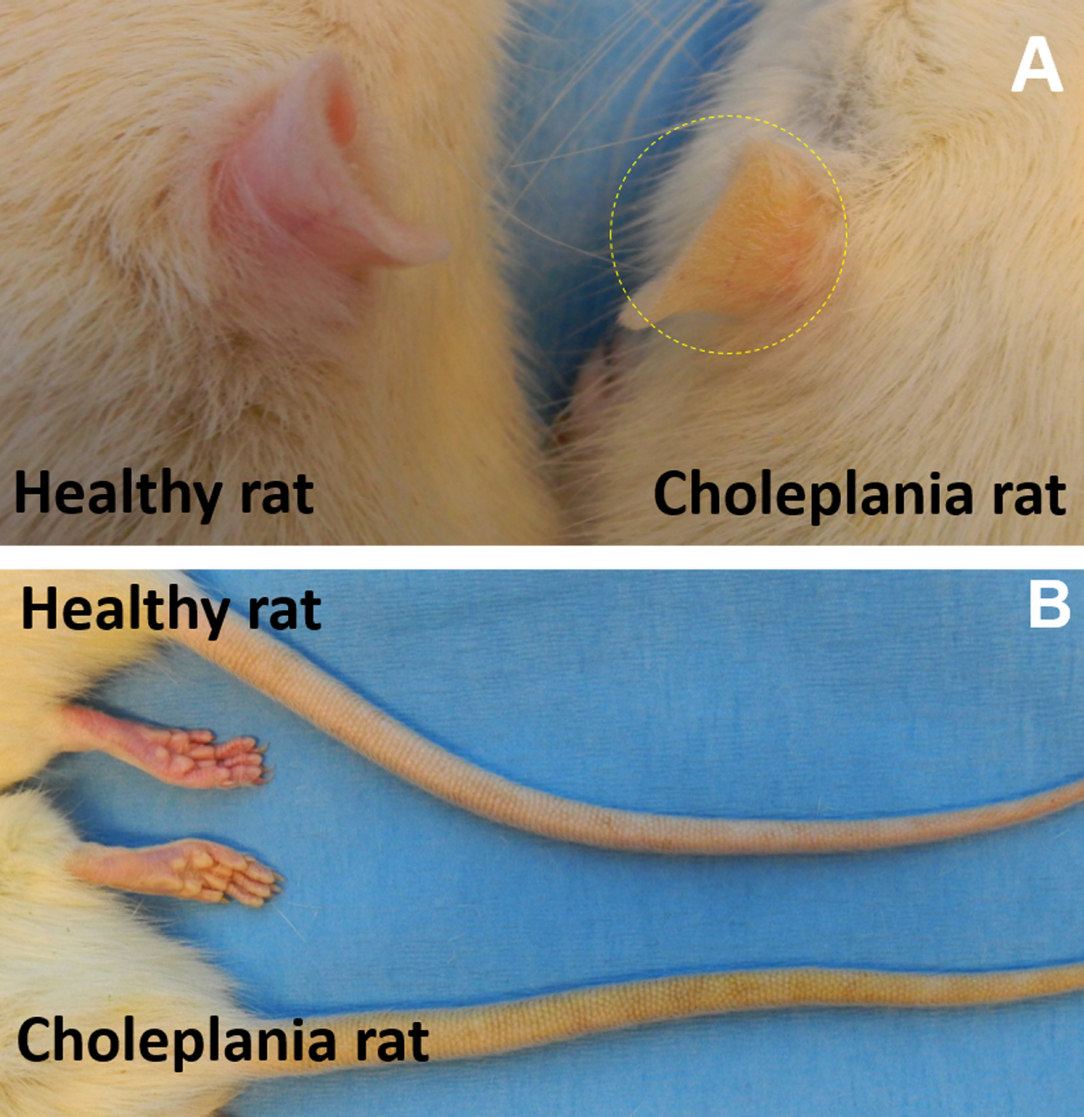
Title A choleplania rat with obstructive jaundice presents yellowish pigmentation of ears (circled on **A**), and tail (**B**), compared to pinkish appearance of a healthy rat.

**Figure 4:**
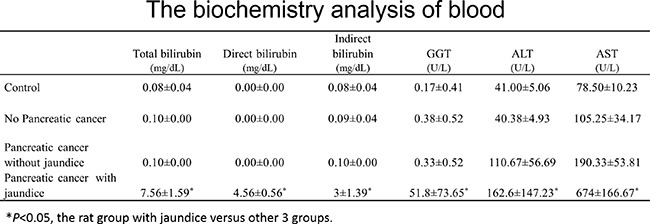
Laboratory biochemistry test demonstrated significant elevation of serum total bilirubin, direct bilirubin, indirect bilirubin, λ-GGT, AST, and AST, compared with the group with no jaundice

Eleven (40.7%) of 27 rats developed jaundice due to biliary obstruction, with typical physical signs of jaundice (Figure 3). Compared with the group with no jaundice, laboratory biochemistry test demonstrated significant elevation of serum total bilirubin (7.56 ± 1.59 mg/dL vs 0.10 ± 0.00 mg/dL), direct bilirubin (4.56 ± 0.56 mg/dL vs 0.00 ± 0.00 mg/dL), indirect bilirubin (3 ± 1.39 mg/dL vs 0.10 ± 0.00 mg/dL), λ-GGT (51.8 ± 73.65 U/L vs 0.33 ± 0.52 U/L), AST ( 162.6 ± 147.23 U/L vs 110.67 ± 56.69 U/L), and AST ( 674 ± 166.67 U/L vs 190.33 ± 53.81 U/L), but there is no significant difference between the size of tumors of the rats with jaundice and without jaundice ( 4.13 ± 0.15 cm^3^ vs 3.97 ± 0.23 cm^3^, *P* = 0.67) (Figure 4).

## DISCUSSION

Pancreatic cancer remains one of the most devastating malignancies in the world [10, 20, 21]. Despite significant improvements in diagnostic imaging modalities and surgical outcome, the prognosis of the disease after the curative surgical resection remains extremely dismal [22]. For the tumors diagnosed at the advanced stage with no chance of surgical resection, systemic chemotherapy and molecular target therapy can only offer modest benefits for prolonging the overall survival and improving the quality of life [10]. Thus, it is imperative to develop alternatives to manage this deadly disease.

One of the primary reasons leading to the lack of efficient therapeutic treatments of this deadly disease is the difficulty in creation of adequate preclinical animal models. These models are crucial for not only studying the underlying mechanisms of pancreatic tumour initiation, growth and dissemination, but also developing new strategies for early detection and effective interventions [14, 23]. Because there are no large animal models with pancreatic cancer so far, the technical verification achieved on normal large animals cannot be translated to clinical application. Currently, the most widely used animal models of pancreatic cancers are created on immunodeficient rodents like nude and SCID mice with either subcutaneous or orthotopic pancreatic cancer xenografts or genetically-modified mice with spontaneous cancers in the pancreas. However, these mouse models with pancreatic cancer are too small to fit in the requirement for developing the image-guided interventional techniques. In this study, we attempted to fill this gap. We have established the protocol of creating a novel rat model with orthotopic pancreatic head cancer, which closely mimics the pathophysiological environment of pancreatic cancer, can be detectable by various molecular imaging modalities, and expected to be useful for developing new image-guided interventional oncology techniques.

The created pancreatic head tumors exhibit the histopathologic features of human pancreatic cancers, such as moderately to well differentiated ductal-type adenocarcinomas, local infiltration to adjacent structures, intraabdominal diffuse metastasis and malignant ascites at the late stage. Most importantly, we established the method of using bioluminescence optical imaging to “screen” the bio-active tumor that emits bioluminescence signals (bright signals) from donor rats. We found that the tumor tissues collected from the bioluminescent active tumor of a donor animal are much more favorable to form pancreatic tumors, compared to the non-bioluminescent (no signal) tumor tissues. These findings indicate the usefulness of optical signals of donor tumors functioning as an indicator for biologically/metabolically active tumors. These results may also explain the reason the success rate of tumor formation is very low in previous creation of such animal models with orthotopic pancreatic cancer, primarily due to unnecessary transplantation of those tumor tissues with no or weak bioactivity.

In addition, the animal model of pancreatic head cancer complicated with obstructive jaundice has the important clinical significance because painless obstructive jaundice is usually the primary sign and symptom of pancreatic head cancer. The rapid accumulation of bilirubin in the circulation can cause severe damages to multiple organs such as the liver, kidney and central nervous system etc. In addition, the timely and effective alleviation of jaundice is the critical prognostic factors of long-term survival of patients. We may use the alleviation of jaundice as the sign to evaluate the therapeutic effect of imaging-guided local treatments, such as intratumoral gene therapy and oncolytic viral therapy, radiofrequency ablation, microwave ablation, and irreversible electroporation ablation.

In this study, we also validated the technical feasibility of using different imaging modalities, including MRI, ultrasound imaging, and optical imaging to image the rat orthotopic pancreatic cancers. Both MRI and ultrasound imaging can be used to characterize the tumors with favorable image quality, which ensure the success of following up the tumor growth, monitoring the therapeutic effect. For *in-vivo* imaging of such animal models with orthotopic pancreatic head cancers, optical imaging may not be an adequate choice, due to the deep location of the pancreatic head tumors that weakens the transmission of bioluminescent signal.

In conclusion, we have successfully established the protocol of using imaging modality to monitor the creation of orthotopic pancreatic head cancer complicated with obstructive jaundice in immunocompetent Lewis rats. Our study indicates that it is necessary to use bioluminescence optical imaging to “screen” the donor tumors, and thereby select those donor tumors with the highest bioactivity for the creation of recipient rat models with orthotopic pancreatic head cancer. The successful creation of these image-detectable animal models with orthotopic pancreatic head cancer may provide a valuable tool for developing novel diagnostic and treatment strategies for pancreatic malignancies.

## MATERIALS AND METHODS

### Cells

We transfected rat pancreatic cancer cells (DSL-6A/C1) with luciferase (Luc)/red fluorescence protein (RFP) /lentivirus gene to create Luc/RFP-positive cells based on the protocol provided by the manufacturer (GeneCopoeia Inc., Rockville, MD, USA). Luc/RFP-positive cells were sorted out using the fluorescence-activated cell sorting technique (Aria II, Becton Dickinson, Franklin Lakes, NJ, USA).

### Animal model creation

The animal protocol was approved by our Institutional Animal Care and Use Committee. We used Lewis rats, weighted 150–200 g (Harlan Laboratories, Livermore, CA, USA), for creating the animal models with orthotopic pancreatic head cancer. The animals were anesthetized with 1–3% isoflurane (Piramal Healthcare, Andhra Pradesh, India) in 100% oxygen. We first created subcutaneous pancreatic cancers in the donor rats. 5 × 10^6^–1 × 10^7^ Luc/RFP cells were subcutaneously injected into right flanks of six rats at 3 spots of each donor rat. Then, we used bioluminescence optical imaging (Bruker Corp., Billerica, MA, USA) to screen the subcutaneous tumors with bioluminescent (bright) signals, which indicated the bio-active or metabolic active tumor tissues of the donor rats. The tumors emitting bioluminescence signal were determined as the donor tissue with the highest viability (ie. the highest biological activity).

**Figure 5:**
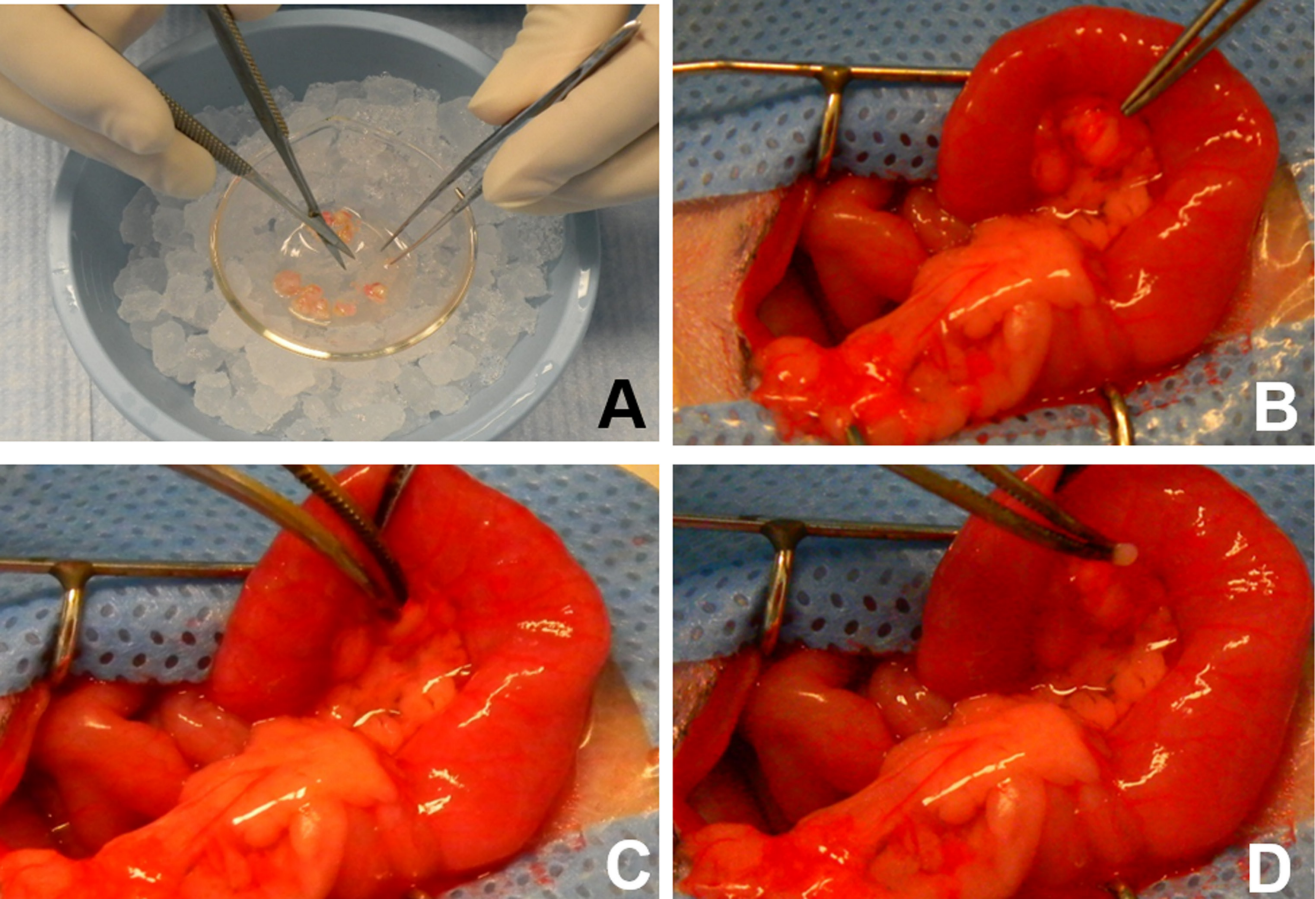
The procedures of inoculating the pancreatic cancer pieces in the pancreatic head of a Lewis rat (**A**) The donor tumor tissue is cut into 1–2 mm^3^ pieces using micro scissors. (**B** and **C**) A small pouch is created in the region of pancreatic head by a blunt dissection. (**D**) Five donor tumor pieces are inserted into to the pouch, followed by a compression using a gelfoam sponge (arrow on D).

The bioluminescent active tumors of the donor rats were excised under sterile conditions once they grew to 10–15 mm in the diameter. We cut the tumor tissues into small pieces of 1 mm^3^ and then transplanted into 52 recipient Lewis rats. For the transplantation, a laparotomy via a median abdominal incision was performed to expose the duodenum, the antrum of the stomach and the pancreatic head. A tiny pouch to accommodate the donor tumor pieces was prepared in the parenchyma of the pancreatic head area with a micro-scissor (RS-5610 VANNAS; Roboz, Rockville, MD). Five tumor fragments were inserted into the pouch and the incision in the pancreatic tissue was compressed by gelfoam sponge for 5 minutes. The pancreas was replaced into the abdominal cavity, and the median incision was closed in two layers with 5–0 absorbable sutures (Figure 5).

### Following tumor growth with bioluminescent optical imaging, ultrasound imaging and MR imaging

Recipient rats underwent optical imaging on a Bruker In-Vivo Xtreme Imaging Systems (Bruker Corp., Billerica, MA, USA). We anesthetized the animals with 1–3% isofluorane delivered in 100% oxygen. Three weeks after the tissue transplantation, each animal was imaged once a week for 4–8 weeks. Bioluminescent images were acquired twenty minutes after intraperitoneal administration of Pierce D-Luciferin (150 mg/Kg) (Thermo Fisher Scientific, Pittsburgh, PA, USA).

For each animal, transcutaneous ultrasound imaging was also performed to follow the tumor growth. Ultrasound imaging (Sonosite Inc, Bothell, WA, USA) was performed to measure the tumor size, with two longest perpendicular axes at X and Y planes, as well as the depth axes defined as Z of each tumor, by using a 6–13 MHz ultrasound transducer. MR imaging was performed using a 3T MRI scanner (Achieva, Philips, Best, Netherland) to follow the tumor growth 4 weeks after the tumor tissue implantation. Animals were placed supine in a 4-chanell wrist coil (Philips, Best, Netherland). For tumor localization, T2-weighted images of 12 slices in coronal orientation were acquired with following parameters: TR/TE, 3000/50ms; field of view (FOV), 100 mm; flip angle, 150; and slice thickness, 3 mm. T1 weighted images of 12 slices in axial orientation were acquired with TR/TE, 55/11.9; FOV, 80 mm; 4 averages; flip angle, 6; and slice thickness, 3 mm. Then, contrast-enhanced MRI was performed by using a transverse T1-weighted fast low angle shot sequence that consisted of one pre-contrast and one postcontrast image obtained 30 seconds after intravenous administration of MR contrast agent (0.2 mmol/kg body weight of Gd-DTPA, Bayer Schering Pharma, Leverkusen, Germany), with the following parameters: TR/TE, 55/11.9ms; FOV, 80 mm; 4 averages; flip angle, 15; slice thickness, 3 mm; FOV, 80 mm; matrix size, 320 × 320; and section thickness, 3 mm.

All animals underwent autopsy after the final MRI. The perpendicular diameters of the primary orthotopic tumor were measured by calipers, and the volume was calculated using the following formula: tumor volume (V) = ¼length × width × depth/2. The adjacent organs, including spleen, stomach, liver (hilus), kidney, retroperitoneum, diaphragm, mesentery loops, and abdominal wall, were examined thoroughly to determine the local tumor infiltration and distant metastases of the tumors. Isolated tumor nodules with no direct anatomic connection to the primary tumor were considered distant metastases.

### Biochemistry test and histology

After the final imaging evaluation, rats were euthanized immediately by respiration of CO2 at a flow of 5 liters per minute for 10 minutes. Blood samples were collected into vacutainer tubes by cardiac puncture with ethylenediaminetetraacetic acid (EDTA) anticoagulant. Laboratory biochemistry test was performed using standard methodologies, including serum albumin, bilirubin (total, direct and indirect), aspartate transaminase (AST), alkaline phosphatase (ALT), gamma-glutamyl transpeptidase (λ-GGT). Tumors were harvested and cryosectioned at 8-μm slices for haematoxylin and eosin staining to confirm the formation of orthotopic pancreatic head cancers.

### Statistics analysis

Statistical software (SPSS, Version 19.0; Chicago, IL, USA) was used for all data analyses. Chi square test (χ^2^ test) was used to compare the tumor formation rate between the animal groups transplanted with the tumor tissues emitting the significant bioluminescence signals and the animal groups with low or invisible bioluminescence signals. Independent student's *t* test was used to compare the serum levels of total bilirubin, direct bilirubin, indirect bilirubin, and tumor size between the animal groups with obstructive jaundice and without jaundice. A *P* value of less than 0.05 was considered significant difference.
